# The purinergic receptor subtype P2Y_2_ mediates chemotaxis of neutrophils and fibroblasts in fibrotic lung disease

**DOI:** 10.18632/oncotarget.16414

**Published:** 2017-03-21

**Authors:** Tobias Müller, Susanne Fay, Rodolfo Paula Vieira, Harry Karmouty-Quintana, Sanja Cicko, Korcan Ayata, Gernot Zissel, Torsten Goldmann, Giuseppe Lungarella, Davide Ferrari, Francesco Di Virgilio, Bernard Robaye, Jean-Marie Boeynaems, Michael R. Blackburn, Marco Idzko

**Affiliations:** ^1^ University Hospital Freiburg, Department of Pneumology, Freiburg, Germany; ^2^ University Hospital RWTH Aachen, Division of Pneumology, Aachen, Germany; ^3^ Department of Biochemistry and Molecular Biology, Houston Medical School, University of Texas, Houston, USA; ^4^ Research Center Borstel, Clinical and Experimental Pathology, Borstel, Germany; ^5^ Department of Physiopathology and Experimental Medicine, University of Siena, Siena, Italy; ^6^ Department of Experimental and Diagnostic Medicine, Section of General Pathology and Interdisciplinary Center for the Study of Inflammation (ICSI), University of Ferrara, Ferrara, Italy; ^7^ IRIBHM and Erasme Hospital, Université Libre de Bruxelles, Brussels, Belgium

**Keywords:** ATP, chemotaxis, neutrophils, nucleotides, pulmonary fibrosis

## Abstract

Idiopathic pulmonary fibrosis (IPF) is a devastating disease with few available treatment options. Recently, the involvement of purinergic receptor subtypes in the pathogenesis of different lung diseases has been demonstrated. Here we investigated the role of the purinergic receptor subtype P2Y_2_ in the context of fibrotic lung diseases.

The concentration of different nucleotides was measured in the broncho-alveolar lavage (BAL) fluid derived from IPF patients and animals with bleomycin-induced pulmonary fibrosis. In addition expression of P2Y_2_ receptors by different cell types was determined. To investigate the functional relevance of P2Y_2_ receptors for the pathogenesis of the disease the bleomycin model of pulmonary fibrosis was used. Finally, experiments were performed in pursuit of the involved mechanisms.

Compared to healthy individuals or vehicle treated animals, extracellular nucleotide levels in the BAL fluid were increased in patients with IPF and in mice after bleomycin administration, paralleled by a functional up-regulation of P2Y_2_R expression. Both bleomycin-induced inflammation and fibrosis were reduced in P2Y_2_R-deficient compared to wild type animals. Mechanistic studies demonstrated that recruitment of neutrophils into the lungs, proliferation and migration of lung fibroblasts as well as IL6 production are key P2Y_2_R mediated processes.

Our results clearly demonstrate the involvement of P2Y_2_R subtypes in the pathogenesis of fibrotic lung diseases in humans and mice and hence support the development of selective P2Y_2_R antagonists for the treatment of IPF.

## INTRODUCTION

Although some progress has been made recently idiopathic pulmonary fibrosis (IPF) is still a devastating disease with few available treatment options, at least partly due to our lack of knowledge about IPF pathophysiology. Currently, it is supposed that the fibrotic response is driven by mediators secreted by abnormally activated alveolar epithelial cells type II after epithelial cell injury. The excessive deposition of collagen and other components of the extracellular matrix eventually results in scarring and destruction of the lung architecture [1–3].

Nucleotides such as adenosine-5′-triphosphate (ATP), uridine-5′-triphosphate (UTP) or uridine-5′-diphosphate (UDP) are released into the extracellular space following tissue injury. Consecutively, elevated levels of extracellular nucleotides have been measured under inflammatory or hypoxic conditions [4–6]. The effects of extracellular nucleotides are mediated via P2 purinergic receptors (P2Rs) which can be subdivided into metabotropic P2Y (P2Y_1_, P2Y_2_, P2Y_4_, P2Y_6_, P2Y_11_-P2Y_14_) and ionotropic P2X receptors (P2X_1_-P2X_7_) [7]. Expression of P2R has been shown on both immune and lung structural cells [5, 8]. Activation of P2Rs is associated with different cellular responses, including migration, release of cytokines or growth factors, production of reactive oxygen species, or apoptosis [7]. In addition, the involvement of specific P2R subtypes in the pathophysiology of different lung disorders has been shown extensively [4, 6, 8–10]. Recently, it has been demonstrated that ATP is released into the extracellular space upon bleomycin administration and that P2X_7_R-deficiency is associated with reduced pulmonary inflammation and fibrosis in different animal models [11, 12]. However, due to the above mentioned widespread expression of purinergic receptors the involvement of more than one receptor subtype is likely.

Functional expression of P2Y_2_ receptors, which are preferably activated by ATP or UTP, has been observed on different inflammatory cells including neutrophils, eosinophils, alveolar macrophages, and dendritic cells [13–16]. Previous studies demonstrated the involvement of this receptor subtype in allergic airway inflammation and chronic obstructive pulmonary disease [6, 16]. However, the precise role of P2Y_2_ receptors in the context of fibrotic lung disease has not been investigated yet.

## RESULTS

### Increased extracellular ATP levels in idiopathic pulmonary fibrosis

**Figure 1:**
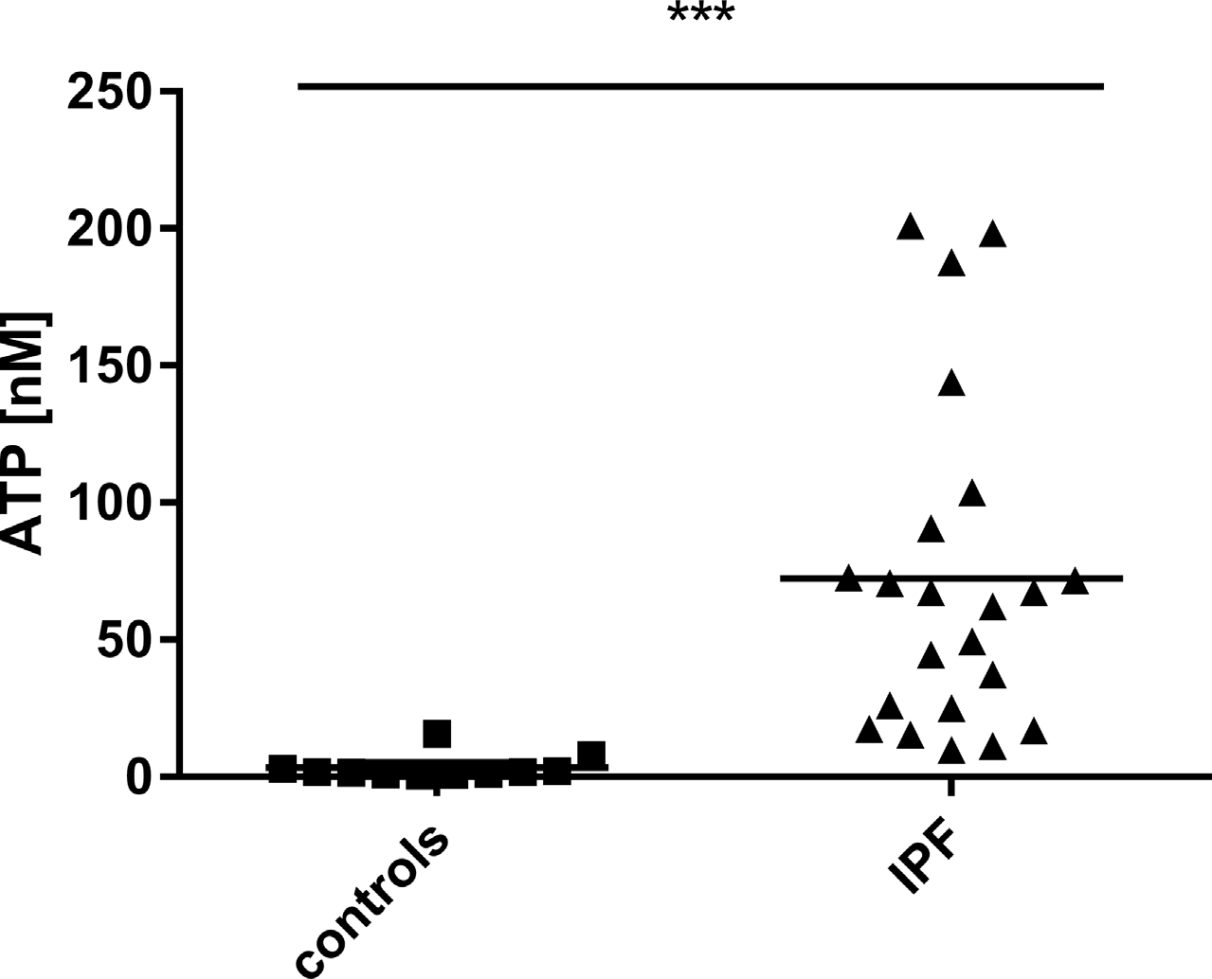
Elevated ATP levels in patients suffering from pulmonary fibrosis ATP levels in BAL fluid obtained during bronchoscopy from patients with IPF (*n* = 22) and healthy volunteers (*n* = 11) were determined using a luminometric assay. ***p < 0.001.

Broncho-alveolar lavage (BAL) fluid obtained during bronchoscopy was used to measure extracellular ATP levels. As shown in Figure 1, ATP levels in the BAL fluid derived from IPF patients were significantly increased compared to healthy subjects (for patient characteristics see Table 1).

**Table 1:** Patient characteristics

	FEV1	TLC	DLCOc	Age	sex	treatment for ILD
1	82	65	43	71	m	no
2	54	56	31	49	m	no
3	77	58	36	89	m	no
4	55	69	N/A	84	m	no
5	94.2	82	94.5	61	m	no
6	72.9	76.4	61.8	73	f	no
7	55	75	N/A	55	m	no
8	76.5	68.3	49.7	82	m	no
9	88	66	50	80	m	prednisone
10	54.7	62.5	17.7	51	m	no
11	39.4	35.1	9	59	m	prednisone
12	86.1	75.3	N/A	86	m	no
13	56.2	58.2	33.1	75	m	prednisone
14	51.9	61.5	52.5	55	m	prednisone
15	65.9	55.5	35.5	73	f	study drug
16	50.4	44.1	31.6	61	m	Prednisone study drug
17	43	62.5	47.8	63	m	no
18	59.9	66.5	29.8	58	m	prednisone
19	41.3	48.8	N/A	65	f	prednisone azathioprine
20	53.1	65.7	39.7	73	f	no
21	68.7	83.3	31.7	88	f	no
22	33.7	37.3	17.2	59	m	prednisone cyclophosphamide

### Functional up-regulation of P2Y_2_R subtypes in idiopathic pulmonary fibrosis

**Figure 2:**
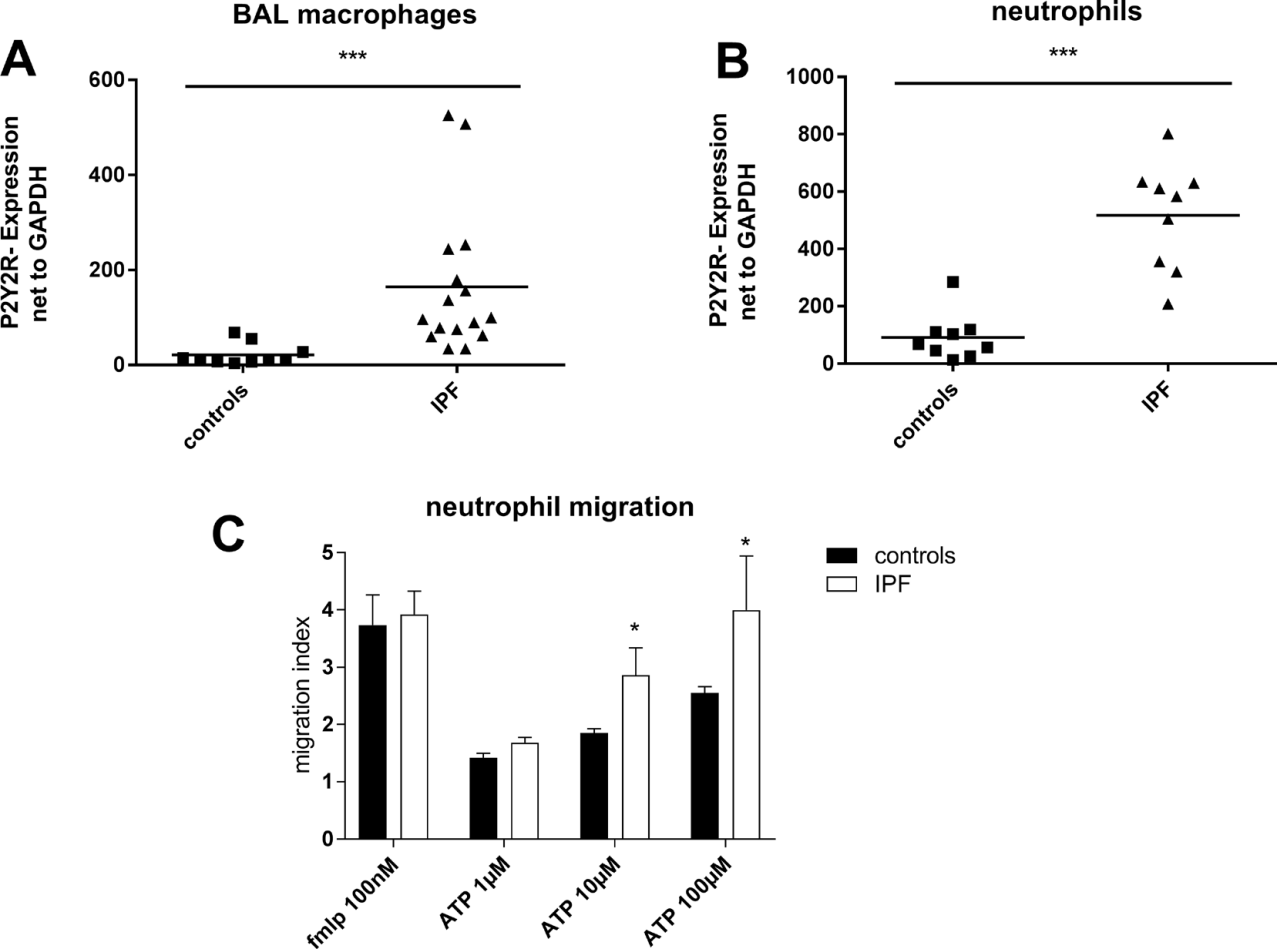
Up-regulation of P2Y_2_R expression in idiopathic pulmonary fibrosis (**A**) Expression of P2Y_2_ receptor subtypes on BAL cells derived from IPF patients (*n* = 16) and healthy individuals (*n* = 10) was determined by quantitative RT-PCR. (**B**) Neutrophils were isolated from whole blood of IPF patients (*n* = 9) and healthy individuals (*n* = 9) and P2Y_2_R expression was measured by quantitative RT-PCR. (**C**) Neutrophils were isolated from whole blood derived from IPF patients (*n* = 5) or healthy volunteers (*n* = 6). Migration in response to ATP or fMLP as a positive control was assessed using the Boyden chamber. *p < 0.05; ***p < 0.001.

A strong up-regulation of P2Y_2_R expression on BAL fluid macrophages and blood neutrophils derived from IPF patients compared to cells isolated from healthy individuals was observed (Figure 2A, 2B). ATP is known as a strong chemoattractant for neutrophils via P2Y_2_ receptors [17]. Hence, the functional relevance of increased P2Y_2_R expression was investigated by migration assay. The chemotaxis towards ATP of neutrophils isolated from IPF patients was stronger compared to neutrophils isolated from healthy individuals (Figure 2C). In line with this finding, ATP levels correlated with the number of leukocytes in the BAL fluid (*r* = 0.70; *p* = 0.03; data not shown).

### Increased extracellular nucleotide levels and P2Y_2_R expression in experimental lung fibrosis

**Figure 3:**
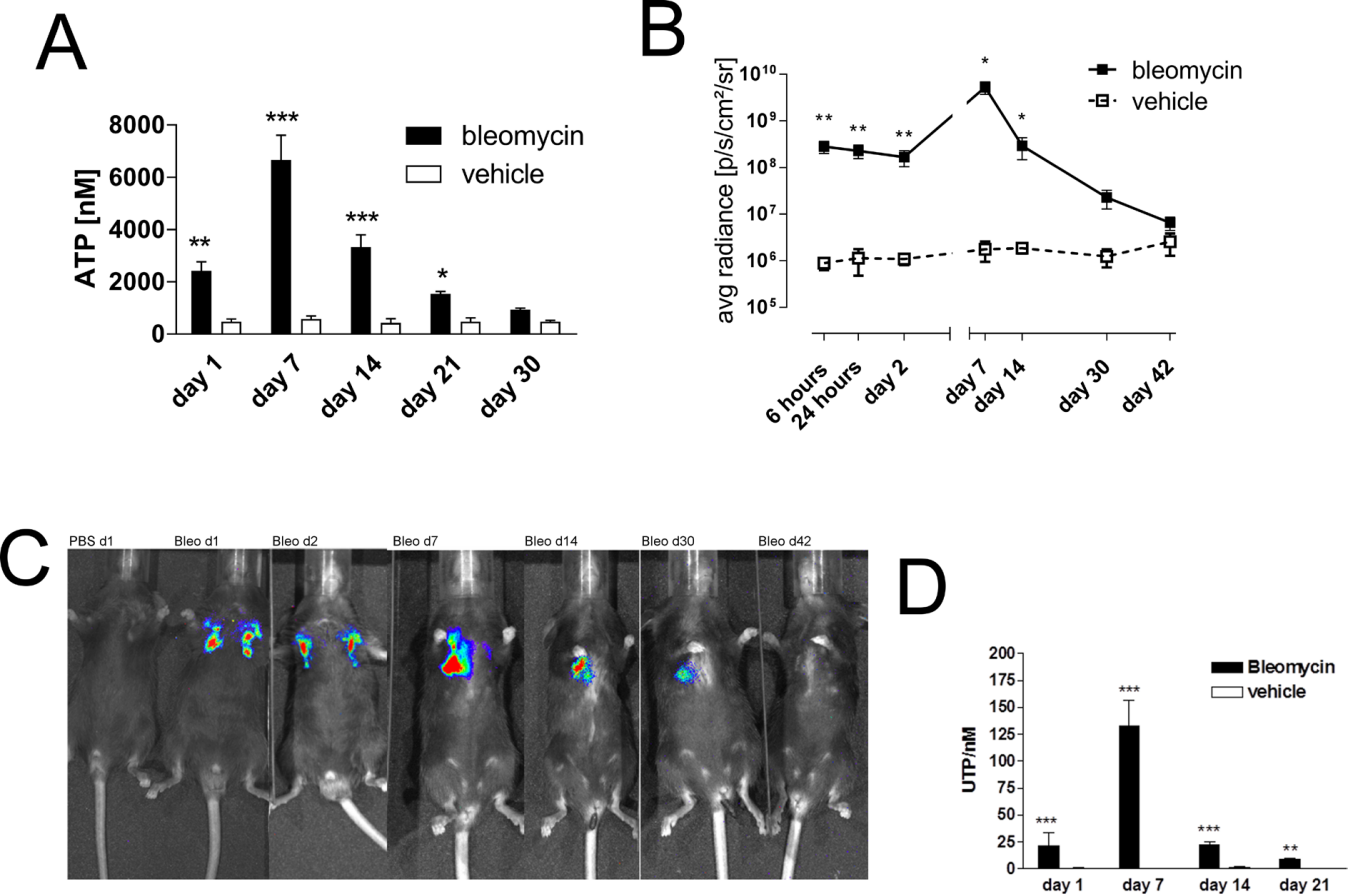
Elevated intrapulmonary nucleotide levels following bleomycin exposure in mice (**A**) Male C57Bl/6 mice received an i. t. injection of BLM or vehicle on day 0. Animals were killed at the indicated time points and ATP levels were measured (*n* = 4–5 per group). (**B**–**C**) Mice received ATP-dependent luciferase-expressing HEK293 cells, followed by an i. t. injection of BLM or vehicle on day 0. Bioluminescence was quantified at the indicated time points (*n* = 3–6 per group). (**D**) Male C57Bl/6 mice received an i. t. injection of BLM or vehicle on day 0. Animals were killed at the indicated time points and UTP levels were measured (*n* = 4–5 per group). *p < 0.05; **p < 0.01; ***p < 0.001.

To confirm the pathophysiological relevance of these results *in vivo* we switched to the well characterized animal model of bleomycin-induced pulmonary fibrosis [18]. Extracellular ATP was significantly increased in the BAL fluid following bleomycin administration and maximal ATP concentration was observed at day 7 with a decline at later time points (Figure 3A), extracellular ATP was significantly increased in the BAL fluid following bleomycin administration and maximal ATP concentration was observed at day 7 with a decline at later time points (Figure 3A). Similar results were obtained when ATP release was visualized *in vivo* by ATP dependent luciferin-induced bioluminescence from HEK293-pmeLUC cells (Figure 3B, 3C) [19]. In addition, BAL fluid UTP levels were also increased in a time-dependent manner after intratracheal bleomycin administration (Figure 3D).

**Figure 4:**
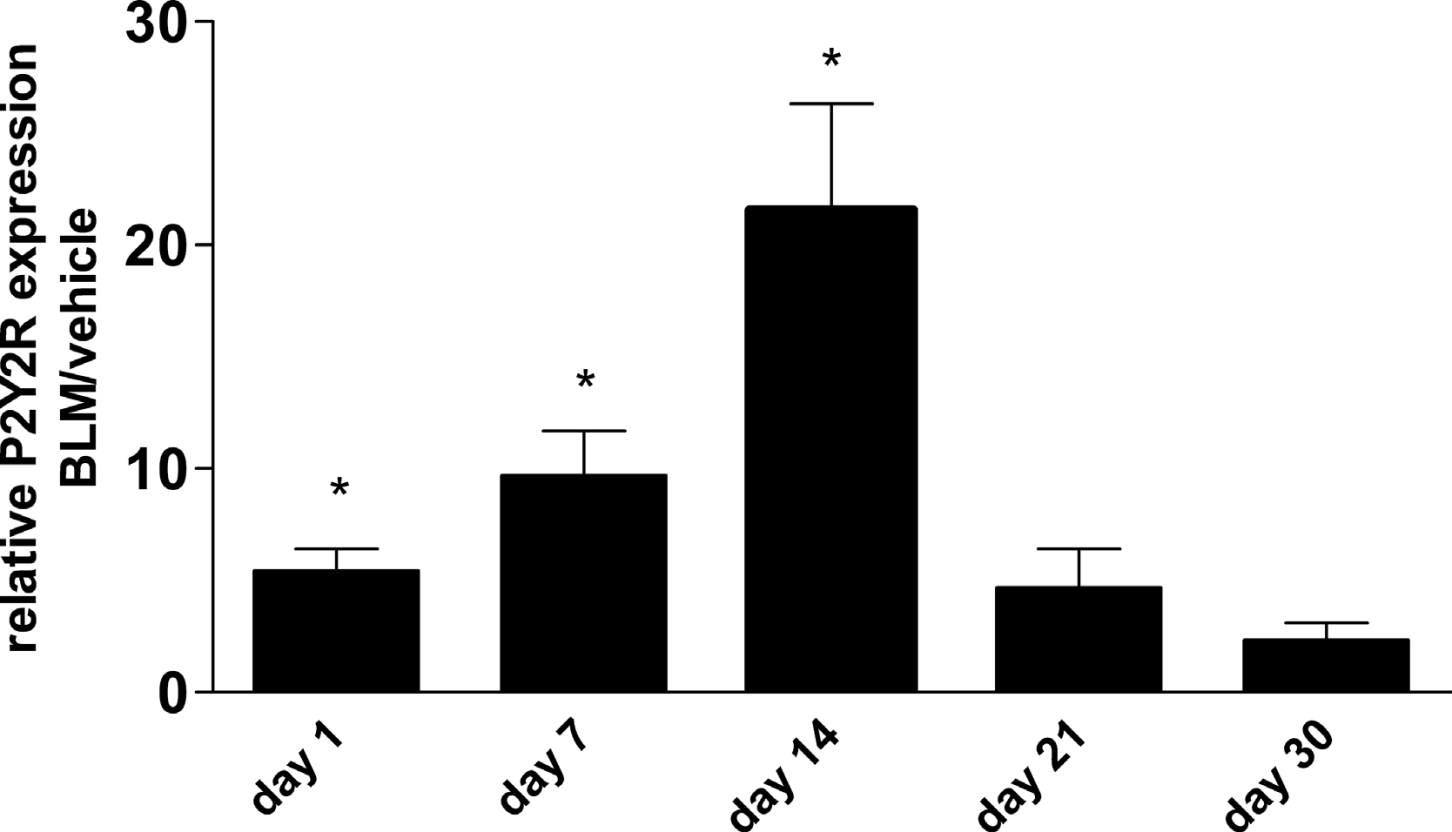
P2Y_2_R expression in bleomycin induced pulmonary fibrosis Male C57Bl/6 mice received an i. t. injection of BLM or vehicle on day 0. Animals were killed at the indicated time points and P2R expression in lung tissue was analysed by quantitative RT-PCR (*n* = 3–5 per group). *p < 0.05.

Apart from extracellular nucleotide levels, there was also an up-regulation of P2Y_2_R expression in whole lung tissue after bleomycin instillation followed by a decline at later time points (Figure 4).

### P2Y_2_R-deficiency is associated with reduced inflammation and fibrosis after bleomycin administration

**Figure 5:**
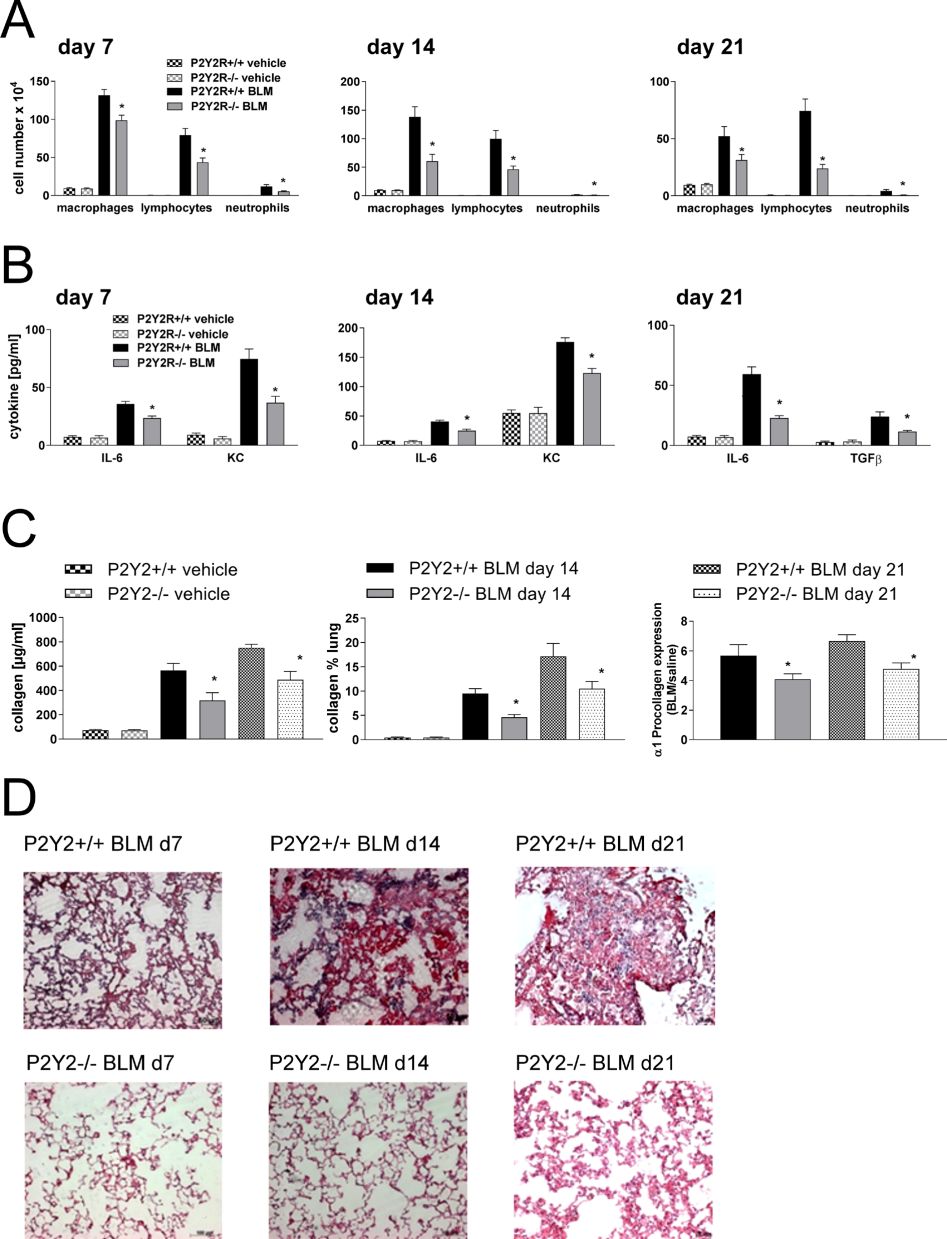
P2Y_2_R-deficient animals are partially protected from bleomycin induced lung injury and fibrosis P2Y_2_R-deficient or wild type mice received an i. t. injection of BLM or vehicle on day 0 and were killed 7, 14 or 21 days later. (**A**) Total and differential cell counts of the BALF were performed. (**B**) BALF cytokines were measured by ELISA. (**C**) Collagen contents were quantified in the BAL fluid by Sircol assay (left) and on histological lung slides (middle). α1 procollagen expression was determined by quantitative real time PCR (right). (**D**) Histological staining of lung slides (H&E). (*n* = 4–14 per group). *p < 0.05 compared to BLM-treated wt animals at the same time point.

We next questioned whether P2Y_2_R-deficiency could alter the course of fibrotic lung disease *in vivo*. Therefore, P2Y_2_R-deficient or wild type animals received an intratracheal injection of bleomycin or vehicle as a negative control at day 0. The degree of inflammation and fibrosis was determined at different time points (day 7, day 14 and day 21 after bleomycin). As shown in Figure 5, pulmonary inflammation was reduced in P2Y_2_R-deficient animals compared to wild type animals at all time points demonstrated by a decreased number of inflammatory cells in the BAL fluid (Figure 5A), decreased concentration of the pro-inflammatory cytokines keratinocyte-derived chemokine (KC) and IL-6 (Figure 5B), and by reduced tissue infiltration by inflammatory cells (Figure 5D). Reduced fibrotic tissue remodelling was confirmed by reduced collagen content in the BAL fluid and on histological lung slides (Figure 5C). Furthermore, BAL fluid levels of the pro-fibrotic cytokine TGF-β (Figure 5B, right panel) and α1 pro-collagen expression (Figure 5C) were significantly lower in P2Y_2_R-deficient animals.

### P2Y*2*R activation is associated with multiple pro-inflammatory and pro-fibrotic effects

**Figure 6:**
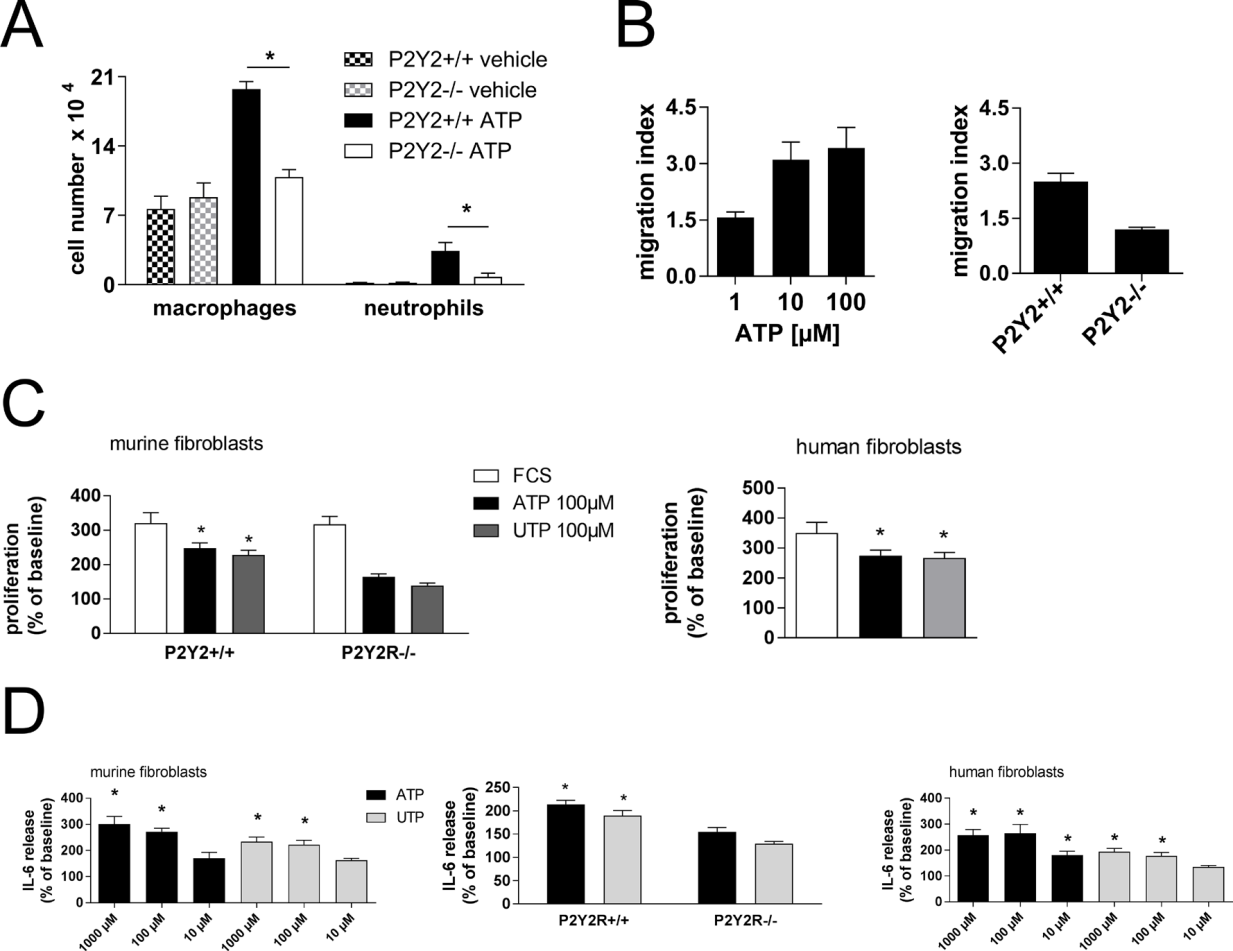
Mechanisms in P2Y_2_R-induced inflammation and fibrosis (**A**) Wild type or P2Y_2_R-deficient (P2Y_2_R−/−) animals received an i. t. injection of ATP. BAL was performed after 24 h, and the number of BALF neutrophils and macrophages was determined. *p < 0.05 compared to ATP-treated wild type animals. (**B**) Primary lung fibroblasts were generated from wild type or P2Y_2_R-deficient animals. Migration towards ATP (100 μM) was analysed via Boyden chamber (*n* = 3 per group). (**C**) Proliferation rate of primary lung murine fibroblasts derived from wild type or P2Y_2_R-deficient animals (left) or human fibroblasts (right) after stimulation with ATP or UTP. (*n* = 3–5 per group). *p < 0.05 compared to vehicle stimulated cells. (**D**) IL6 release of primary murine lung fibroblasts (left and middle) or human lung fibroblasts (right) stimulated with different nucleotides was measured by ELISA (*n* = 3–7 per group). *p < 0.05 compared to vehicle-stimulated fibroblasts.

Recruitment of neutrophils into the lungs has been demonstrated to play a pivotal role in the pathogenesis of fibrotic lung disease [20]. Naïve wild type and P2Y_2_R-deficient animals received an intratracheal injection of ATP and the number of inflammatory cells in the BAL fluid was assessed after 24 h. As shown in Figure 6A, ATP increased the numbers of neutrophils and macrophages in the lungs of wild type animals whereas no changes were seen in P2Y_2_R-deficient mice.

Increased migration of lung fibroblasts to the site of tissue injury is essential for the development of pulmonary fibrosis [1]. ATP induced migration of fibroblasts isolated from wild type but not from P2Y_2_R-deficient animals (Figure 6B).

Proliferation of lung fibroblasts is also crucial for the pathogenesis of pulmonary fibrosis [1]. ATP and UTP increased the proliferation rate of both primary murine and human lung fibroblasts (Figure 6C). The involvement of P2Y_2_R subtypes was confirmed by experiments with fibroblasts derived from P2Y_2_R-deficient animals (Figure 6C, left).

The importance of the cytokine IL6 in the context of tissue remodelling has been demonstrated previously [21]. Stimulation of both murine and human lung fibroblasts with ATP or UTP resulted in a dose-dependent increase in IL6 production (Figure 6D). The increase in IL6 secretion was weaker but not absent in fibroblasts derived from P2Y_2_R-deficient animals (Figure 6D, middle).

## DISCUSSION

Signalling via purinergic receptors, e. g. P2Y_2_R subtypes, has been demonstrated to play an important role in the pathogenesis of various inflammatory disorders including bronchial asthma and COPD [7]. In this manuscript we investigated the involvement of extracellular nucleotides and the purinergic receptor P2Y_2_ in the pathogenesis of idiopathic pulmonary fibrosis in humans and in the animal model of bleomycin induced lung fibrosis.

We found elevated ATP concentrations in the BAL fluid in patients suffering from IPF compared to healthy controls. Additionally, a time dependent increase in BAL fluid ATP and UTP levels, peaking at day 7 after bleomycin administration in mice was observed. These data are in contrast to a previous study claiming that BAL fluid ATP levels are only increased very early (day 1) after bleomycin application in mice [11]. A possible explanation might be that Riteau and co-workers used a higher dosage of bleomycin [11]. Nevertheless, we think that our animal model with a chronic increase in extracellular ATP is much closer to the situation in humans with chronically elevated BAL fluid ATP levels in IPF patients.

Bleomycin-induced pulmonary fibrosis is known to be self-limiting with maximal fibrotic response around day 14 and a resolution of the fibrotic changes at later time points [18]. Hence, higher nucleotide levels in the extracellular space are paralleled by increased inflammation and fibrosis, in accordance with previous studies demonstrating increased extracellular ATP levels in inflammatory lung diseases such as bronchial asthma or COPD [4–6, 22]. Thereby, ATP release can occur via lytic and non-lytic pathways, e. g. via pannexins or connexin hemichannels or via exocytosis [7].

Apart from elevated extracellular nucleotide levels, an increased expression of P2Y_2_ receptors on cells derived from IPF patients and in the lung tissue of animals with bleomycin induced pulmonary fibrosis was observed. Of note in the animal model, maximal P2Y_2_R expression was found around day 14 when the maximal fibrotic response can be expected. The functional relevance of increased P2Y_2_ receptor expression on blood neutrophils was demonstrated by increased chemotaxis in response to ATP. In line with previous studies, the functional relevance was further supported by the finding that the ATP-induced recruitment of neutrophils into the lungs was highly dependent on P2Y_2_ receptor expression [6, 17].

P2Y_2_R-deficient animals were found to be partially protected from bleomycin-induced pulmonary inflammation and fibrosis. Whereas the involvement of P2Y_2_ receptors in the pathogenesis of different inflammatory disorders (e. g. bronchial asthma, COPD or hepatitis) has been demonstrated previously, much less is known about the role of this receptor subtype in tissue remodelling and fibrosis [6, 16, 23–25]. The effects following P2Y_2_R-activation can be attributed to different mechanisms. Intrapulmonary ATP levels were increased in both IPF patients and animals after intratracheal bleomycin administration paralleled by increased P2Y_2_R expression. Consecutively, given the fact that P2Y_2_ receptors are essential for nucleotide-induced migration of neutrophils, elevated ATP levels in the alveolar space might lead to an influx of blood neutrophils into the lungs during the initiation and progression of fibrotic lung disease [6, 17]. Though the exact role of neutrophils in the pathophysiology of IPF is still a matter of debate, the release of proteolytic enzymes, reactive oxygen species, and profibrotic cytokines by neutrophils can contribute to tissue destruction and fibrosis [26–28]. In accordance, an increased percentage of neutrophils in the BAL fluid has been shown to be a predictor of early mortality in IPF [29]. Activation, proliferation and migration of lung fibroblasts eventually lead to enhanced deposition of extracellular matrix components [28]. Our results clearly show that P2Y_2_R activation is associated with enhanced proliferation and migration of primary lung fibroblasts. Interestingly, nucleotides released upon tissue injury have been demonstrated to be involved in dermal wound healing and cardiac fibrosis via P2Y_2_R subtypes [24, 25]. Finally, P2Y_2_ receptor activation increased IL-6 secretion by lung fibroblasts, a cytokine linked with tissue remodelling and fibrosis apart from its well characterized role in acute inflammation [8, 21, 30].

However, it is very likely that even more mechanisms might be of importance in this context. Alveolar macrophages have been found to play a pivotal role in the pathogenesis of fibrotic lung diseases. Interestingly, P2Y_2_ receptor activation on this cell type has been shown to be associated with chemotaxis and secretion of pro-inflammatory cytokines [31–33].

In summary, we were able to demonstrate that the purinergic receptor subtype P2Y_2_R contributes to the pathogenesis of pulmonary fibrosis via different mechanisms including recruitment of neutrophils as well as migration, proliferation, and cytokine secretion of lung fibroblasts. Hence, targeting P2Y_2_R might be a new treatment option for idiopathic pulmonary fibrosis.

## MATERIALS AND METHODS

### Patient materials

Broncho-alveolar lavage (BAL) fluids were collected from patients undergoing bronchoscopy during the diagnostic workup of IPF or from healthy volunteers. IPF was diagnosed according to published criteria [34]. The study was approved by the local ethics committee (Ethikkommission des Universitätsklinikums Freiburg).

### Animals

P2Y_2_R-deficient and wild type animals (both on C57BL/6 background) were bred at the University Freiburg. All experiments were approved by the local animal ethics committee (Regierungspraesidium Freiburg).

### Bleomycin model of pulmonary fibrosis

Male C57BL/6 or P2Y_2_R-deficient animals were anaesthetized and received an i.t. injection of bleomycin (80 μl; 0.5mg/ml). Animals were killed at different time points via i. p. injection of pentobarbital (5 mg in 0.5 ml). BAL was performed with 3 × 1 ml of Ca^2+^ and Mg^2+^ free PBS supplemented with 0.1 mM sodium EDTA, followed by lung resection and storage in OCT freezing medium. BAL cells were counted, differential cell counts were done by FACS analysis, as described previously [35]. Frozen lung sections were stained with hematoxylin / eosin for histological analysis.

### Mediator measurements in BALF

ATP levels in BALF were measured using ATPLite kit (Perkin Elmer), as previously described [4]. BALF cytokine contents were determined by ELISA (R&D Systems, Minneapolis, USA), as described by the manufacturer. BALF collagen content was measured by Sircol assay (Biocolor, Carrickfergus, U.K.).

### Collagen quantification in histological lung slides

Frozen lung sections were incubated in picrosirius red solution (0, 2 gr of Picosirius Red diluted in 100 ml of 1, 2% picric acid, both Sigma-Aldrich), for one hour. After washing with water, tissue sections were stained with hematoxylin for 5–10 seconds. Slides were washed with running tap water and dehydrated in 70%, 90% and absolute ethanol, followed by xylene. Entellan (Merck) was used to mount the coverslip. Images were obtained using Axio Lab.A1 microscope (Zeiss) with 200 × magnification and AxioCam ICc1 (Zeiss). Collagen quantification was made with ImageJ.

### PCR

Total RNA from human or murine lung tissue was isolated using RNeasy mini-kits (Qiagen, Hilden, Germany) according to the manufacturer's recommendations followed by reverse transcription using Stratascript reverse transcriptase (Stratagene, La Jolla, CA) and random primers (Invitrogen, Karlsruhe, Germany). Quantitative PCR was performed with Taqman Universal PCR Mastermix (Applied Biosystems, Foster City, CA) and pre-formulated primers and probe mixes (Assay on Demand; Applied Biosystems). PCR was performed on a thermal cycler (iCycler; Bio-Rad, Hercules, CA). PCR amplification of the housekeeping gene encoding glyceraldehyde 3-phosphate dehydrogenase was performed during each run for each sample to allow normalization between samples.

### *In vivo* detection of extracellular ATP release

For this imaging, 2 × 10^6^ cells with membrane-targeted luciferase (PME cells) were injected intravenously via tail vein and recipient mice were examined consecutively after bleomycin administration [19, 36].

### *In vitro* migration of lung fibroblasts or blood neutrophils

Blood neutrophils from whole blood of control subjects or IPF patients as well as human and murine fibroblasts were isolated as described previously [5, 37].

Migration was investigated using transwell chambers (Corning, Lowell, USA). Nucleotides or vehicle were added into the lower compartment wells. Cells (5 × 10^4^ cells/well) were added to the upper compartment and incubated at 37°C for 3 h min in a humidified atmosphere; cells in the lower compartment were counted. Results are shown as chemotactic index, calculated as the number of cells in the lower chamber containing the different stimuli divided by the number of cells in the chamber containing medium alone.

### Proliferation of lung fibroblasts

Fibroblasts (2 × 10^5^ cells/well) were seeded into cell culture plates and stimulated with the indicated nucleotides or vehicle. Higher concentrations of FCS (20%) were used as a positive control. After 48 h cells were trypsinized and counted. Results are shown as proliferation index, calculated as the number of nucleotide-stimulated cells divided by the number of vehicle treated cells.

## Abbreviations

ATP: adenosine-5′-triphosphate
BAL: broncho-alveolar lavage
COPD: chronic obstructive pulmonary disease
IL: interleukin
IPF: idiopathic pulmonary fibrosis
UTP: uridine-5′-triphosphate
